# Low-Dose Radiotherapy for Benign Achillodynia: A Single Center Experience With Prospective Follow-Up

**DOI:** 10.7759/cureus.110891

**Published:** 2026-06-15

**Authors:** Hongjian Tang, Olaf Büttner, Daniel R Zwahlen, Paul Windisch

**Affiliations:** 1 Department of Radiation Oncology, Cantonal Hospital Winterthur, Winterthur, CHE; 2 Departments of Orthopedics and Traumatology, Klinik Impuls, Wetzikon, CHE; 3 Department of Radiation Oncology, University Hospital of Bern, Bern, CHE

**Keywords:** achilles tendinopathy, achillodynia, ldrt, low-dose radiation therapy, tendinopathy

## Abstract

Purpose

To evaluate the effectiveness and safety of low-dose radiotherapy (LDRT) for refractory achillodynia and to examine whether pain response differs by pretreatment pain duration or common dose and fractionation schedules.

Methods

Consecutive patients treated for Achilles tendinopathy at a single institution between 2020 and January 2026 were identified and contacted in early 2026 for updated outcome and toxicity assessment. Pain was assessed using a 0 to 10 visual analog scale (VAS) before radiotherapy and during follow-up. Treatments were delivered two to three times weekly according to German Society for Radiation Oncology (DEGRO)-consistent schedules. Response, paired VAS change, toxicity, and subgroup differences were analyzed.

Results

Seventy-one patients received radiotherapy for 81 treated feet, including 10 bilaterally treated patients. Median baseline VAS was 7, and most patients had undergone prior conservative therapies. The most common schedule was 6 × 1 Gy. At a median follow-up of 3 months after the last radiotherapy course, 79% (n = 64) of feet responded, 13.6% (n = 11) did not respond, and 7.4% (n = 6) were lost to follow-up. Among 61 feet with paired VAS data, the median pain improvement was 4 points. No increased pain or treatment-related toxicity was reported. Pain improvement did not significantly differ by pain duration of ≤12 versus >12 months or by 6 × 1 Gy versus 8 × 0.5 Gy.

Conclusion

LDRT provided meaningful pain relief without observed toxicity in this real-world cohort. These findings support LDRT as a potential option after failed, contraindicated, or poorly tolerated conservative treatment with an effect size similar to what has been reported for fasciopathies in randomized controlled trials.

## Introduction

Achilles tendinopathy or achillodynia can be viewed within the broader group of periarticular soft-tissue disorders, which are increasingly understood as overlapping disorders of the enthesis, tendon, and peritendinous structures driven by repetitive microtrauma, mechanical overload, degenerative change, and low-grade inflammation [1-3]. Within this setting, low-dose radiotherapy (LDRT) has been described as an effective option for refractory symptoms that are unresponsive to, or contraindicated for, conservative management [1].

A biological rationale for this approach is provided by translational data showing that LDRT can induce systemic immune modulation, including a shift from CD8+ to CD4+ T cells, reduced dendritic cells, and lower inflammatory cytokine levels [4].

Consistent with this concept, the guideline by the German Society for Radiation Oncology (DEGRO) recommends single fractions of 0.5 to 1.0 Gy and total doses of 3.0 to 6.0 Gy per series, applied two to three times per week, for degenerative-inflammatory diseases when simple and non-invasive methods have not provided persistent success [5].

For achillodynia specifically, prospective evidence is centered mainly on the Erlangen randomized dose-optimization trial, its long-term follow-up, and a broader mixed-indication quality assessment that included 46 patients with achillodynia among 703 evaluable cases [2,3,6]. In the randomized trial, overall response rates were 84% immediately after radiotherapy and 88% at six weeks, with no significant difference between the 0.5 Gy and 1.0 Gy dose levels. Long-term evaluation of the same cohort showed early, delayed, and long-term response rates of 84%, 88%, and 95%, respectively, again without a significant difference between dose levels. In the broader prospective quality assessment, long-term efficacy was significantly better than immediate post-treatment efficacy for achillodynia, and no side effects were observed overall.

Against this background, the present study seeks to add further clinical outcome data on both pain response and toxicity after LDRT for Achilles tendinopathy. In addition, the goal was to publish patient-level data, including pretreatment variables such as pain duration and prior therapies, to enable future aggregation, e.g., for the development of scores to predict response to LDRT.

## Materials and methods

Study design

All sequential patients treated at our institution for Achilles tendinopathy between 2020 and January 2026 were retrieved from the treatment management system and contacted via phone in early 2026 to obtain recent outcome and toxicity results. While providers were encouraged to provide imaging data as part of their initial referral, this was not a requirement.

Treatments had been conducted by different providers at the two sites of our institution using both kilo- and megavoltage radiation, depending on resource availability and provider preference. Treatments were delivered two to three times per week using different doses and fractionation schedules in accordance with current DEGRO recommendations. Two weeks after the conclusion of a course, a first follow-up visit was completed, and the patient was instructed to contact the institution if they did not deem the pain response within the next eight to twelve weeks to be sufficient. In that case, the treatment was repeated. Patients who had recurrent pain after a minimum of one year were eligible for a third series.

Pain severity was assessed using a Likert scale (no response, partial response, near complete response, and complete response) as well as the visual analog scale (VAS) with 0 points indicating no pain and 10 points indicating maximal pain prior to radiotherapy and at every follow-up visit. Prior to treatment initiation, patients were also asked to provide information on therapies they had previously undergone for their Achilles tendinopathy. 

Ethical considerations

The study was conducted in accordance with the Declaration of Helsinki and approved by the responsible Ethics Committee (BASEC 2020-02112). Patients signed a general consent form authorizing the use of their data for research purposes.

Statistical analysis

Data analysis was performed in Python (v. 3.13.7) using the pandas (v. 3.0.0) and matplotlib (3.10.8) packages. Between-group comparisons of VAS improvement were assessed with two-sided Mann-Whitney U tests, with effect sizes reported as Hodges-Lehmann median pairwise differences and approximate 95% confidence intervals derived from the tie-corrected Mann-Whitney variance; 95% confidence intervals for linear regression coefficients were calculated from the residual standard error using the t distribution. For the analysis of response by prior pain duration, a 12-month cut-off was chosen as this constituted a pragmatic separation of recent pain (e.g., after one to two cycles of physiotherapy and other conservative approaches) and chronic pain.

## Results

The patient, treatment, and follow-up characteristics are presented in Table 1 and Table 2.

**Table 1:** Patient and treatment characteristics RT - radiotherapy; NSAID - non-steroidal anti-inflammatory drugs; VAS - visual analog scale

Characteristic	n/N (%)
Sex	
Male	32/71 (45.1%)
Female	39/71 (54.9%)
Prior therapy	
Painkillers prior to RT	30/71 (42.3%)
Physiotherapy	46/71 (64.8%)
Shockwave	31/71 (43.7%)
Topical NSAID	15/71 (21.1%)
Insoles	17/71 (23.9%)
Injection	9/71 (12.7%)
Autologous Blood	5/71 (7.0%)
Ultrasound	5/71 (7.0%)
Acupuncture	2/71 (2.8%)
Surgery	2/71 (2.8%)
Massage	4/71 (5.6%)
Leeches	3/71 (4.2%)
Radiation technique	
Kilovoltage (kV)	57/81 (70.4%)
Megavoltage (MV)	24/81 (29.6%)
Dose and fractionation schema	
3.0 Gy = 6×0.5 Gy	2/81 (2.5%)
4.0 Gy = 8×0.5 Gy	19/81 (23.5%)
6.0 Gy = 6×1.0 Gy	59/81 (72.8%)
7.0 Gy = 7×1.0 Gy	1/81 (1.2%)
Response	
Complete	28/81 (34.6%)
Near-complete	9/81 (11.1%)
Partial	27/81 (33.3%)
None	11/81 (13.6%)
Lost to follow up	6/81 (7.4%)
Number of courses	
1 course(s)	52/81 (64.2%)
2 course(s)	25/81 (30.9%)
3 course(s)	4/81 (4.9%)

**Table 2:** Patient and treatment characteristics RT - radiotherapy; VAS - visual analog scale

Characteristic	N	Mean	Median	IQR	Range
Age	71	65.2	65	60.0 – 73.0	34.0 – 84.0
Pain duration prior to RT (months)	75	29.5	12	6.5 – 24.0	2.0 – 360.0
Follow up (months)	78	8.9	3	2.0 – 8.8	0.5 – 72.0
Pain prior to RT (VAS)	64	6.6	7	5.0 – 8.0	1.0 – 10.0
Pain at last follow up (VAS)	67	1.9	1	0.0 – 3.5	0.0 – 10.0

Seventy-one patients were treated at our institution between 2020 and 2026. Ten patients were treated bilaterally, yielding 81 treated feet in total. 54.9% of patients were female (n = 39) and 45.1% male (n =32). Median and mean age were 65 years (IQR 60-73 years).

Median and mean pain duration prior to radiotherapy were 12 and 29.5 months, respectively (IQR 6.5-24 months). Most patients had undergone a variety of conservative treatment approaches, the most frequent ones being physiotherapy (64.8%, n = 64) and shockwave therapy (43.7%, n = 31). 42.3% (n = 30) of patients reported taking painkillers regularly prior to the radiation therapy start. Two patients had received surgery for Achilles tendinopathy in the past. Median and mean pain scores prior to starting radiotherapy were 7 and 6.6, respectively (IQR 5-8). 

Treatment was delivered using kilovoltage (kV) x-rays for 70.4% (n = 57) and megavoltage x-rays for 29.6% (n = 24) of feet. The most common prescriptions were 150 kV prescribed to 0-1 cm depth (35.8%, n = 29), followed by 100 kV to 0-2 cm depth (28.4%, n = 23). The most commonly used schema was 6 x 1 Gy (72.8%, n = 59), followed by 8 x 0.5 Gy (23.5%, n = 19), each given two to three times per week. Most feet received only one course of radiation (64.2%; n = 52). A second and third course were necessary in 30.9% (n = 25) and 4.9% (n = 4), respectively. 

**Figure 1:**
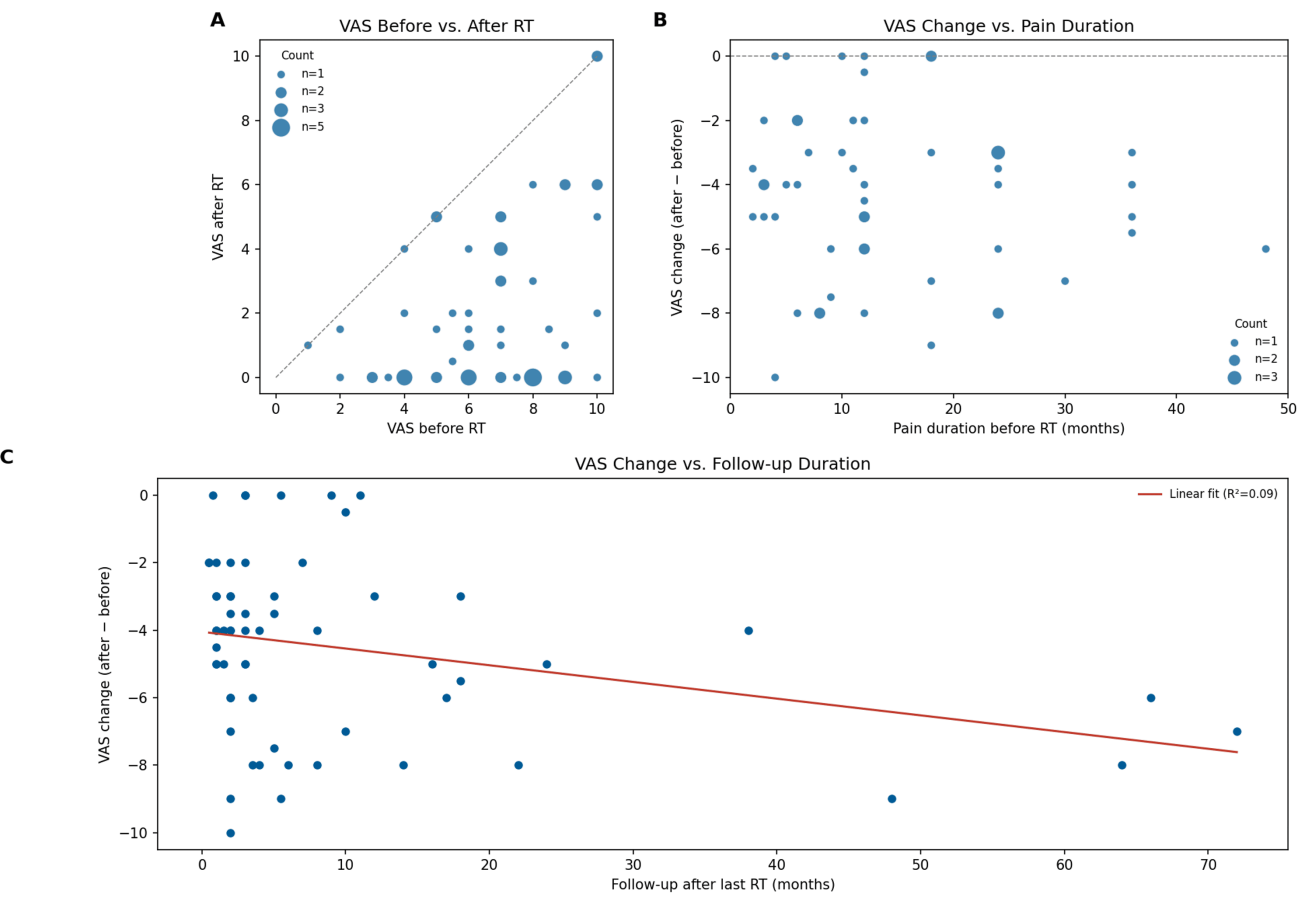
Pain severity A) Scatterplot of pain severity based on pain prior to radiotherapy (x-coordinate) and at the last radiotherapy follow-up (y-coordinate). One dot may represent up to five feet, with larger dots representing more feet, and dots on the dashed line indicate no change in pain from before to after radiotherapy; B) Scatterplot of the change in pain severity by pain duration of pain prior to radiotherapy. One dot may represent up to three feet; C) Scatterplot of the change in pain severity by follow-up duration after the last radiotherapy course. The red line indicates the fit of a linear model with a slope of -0.05 VAS per month (VAS/month 95% CI -0.091 to -0.008). VAS - visual analog scale; RT - radiotherapy

The median and mean duration of follow-up after the last course of radiation were 3 and 9 months, respectively (IQR: 2-9 months). At the last follow-up, a response was reported for 79% of feet (n = 64). For 13.6% of feet (n = 11), there was no response, and 7.4% (n = 6) were lost to follow-up. For the 61 feet where complete pre- and posttreatment pain scores were available, pain had improved by a median of four points (mean: 4.5, IQR 3-6). Changes in pain scores by baseline conditions and length of follow-up are depicted in Figure 1. No patient reported increased pain after the last course of radiotherapy, and no toxicities were reported at any time during follow-up.

We did not find a significant difference in the pain scores between patients who had ≤ 12 months of pain prior to radiotherapy (n = 34, median improvement = 4) vs. those who had > 12 months of pain (n = 26, median improvement = 5, Hodges-Lehmann VAS-delta difference -1.0, approximate 95% CI -2.5 to 0.5, p = 0.20). Likewise, we did not find a significant difference between the two most common dose and fractionation schemas 6 x 1 Gy (n = 43, median improvement = 5) and 8 x 0.5 Gy (n = 16, median improvement 4, Hodges-Lehmann VAS-delta difference 1.0, approximate 95% CI -1.0 to 3.0, p = 0.288).

## Discussion

In this single-center cohort, low-dose radiotherapy was associated with meaningful symptom relief in a heavily pretreated population with chronic achillodynia. At the last follow-up, 79% of treated feet were classified as responders, nearly half had complete or near-complete relief, the median paired VAS improvement was four points, and no patient reported worsening pain or treatment-related toxicity. Given that many patients had already received physiotherapy, shockwave therapy, and regular analgesics, these data suggest that LDRT can provide clinically relevant benefit in patients whose symptoms remain burdensome despite standard conservative care.

These findings are broadly in line with the existing achillodynia literature. In the Erlangen randomized dose-optimization trial, Ott et al. reported overall response rates of 84% directly after treatment and 88% at six weeks, with no significant difference between 0.5 Gy and 1.0 Gy fractions [2]. In the long-term follow-up of the same cohort, response rose to 95% at a median of 24 months, again without a dose-level difference [3]. Micke et al. likewise reported improved long-term compared with immediate response in achillodynia, with 88.9% good response at follow-up among evaluable patients and no observed side effects [6]. Against that background, the 79% response observed here appears plausible and may even underestimate eventual benefit, because the median follow-up after the last course in the present study was only three months, and repeat treatment was allowed in insufficient responders. This is supported by the negative slope of the linear model from Figure 1c, i.e., better pain response with increased follow-up time.

Our comparison between 6 x 1 Gy and 8 x 0.5 Gy that showed no difference in pain response is directionally consistent with prior dose-optimization work and with DEGRO guidance that low-dose schedules in the range of 0.5 to 1.0 Gy per fraction and 3 to 6 Gy per series are reasonable for painful degenerative-inflammatory disorders after failure of simple non-invasive measures [2,5,7]. The lack of a significant association between shorter versus longer pain duration and VAS improvement should be interpreted cautiously because the study was obviously not powered for this analysis, but it does suggest that prolonged symptom duration alone should probably not be viewed as a contraindication to LDRT.

At the same time, these results should be positioned appropriately within the broader Achilles tendinopathy literature. Contemporary reviews and guidelines continue to place structured, progressive tendon loading and other conservative measures at the center of first-line management, while comparative evidence among active nonoperative options remains heterogeneous and often uncertain [8-10]. This matters because the present cohort was clearly not an untreated population: most patients (94%) had already received prior therapies, and the study therefore speaks most directly to a refractory clinical setting. In that context, the observed pain relief is particularly relevant. The fact that about one-third of feet required a second or third radiation course also underlines a practical point from earlier RT series, that appropriate expectation management is important [6,11].

Several strengths support the value of the present study. For a relatively niche indication, the cohort is sizable. The study reflects consecutive real-world practice across two institutional sites, reports both categorical response and absolute VAS change, includes pretreatment variables such as symptom duration and prior therapies, and actively assessed later outcome and toxicity rather than restricting evaluation to the immediate post-treatment period. The inclusion of both kilovoltage and megavoltage treatments also increases practical applicability, by supporting the notion that the observed benefit is not tied to a single platform or a narrowly standardized workflow.

Important limitations remain. This was a single-center, non-randomized study without a control group, so natural history, regression to the mean, placebo effects, and the influence of ongoing co-interventions cannot be excluded. While ongoing co-interventions could have improved the efficacy of radiotherapy as a whole, and we actually instruct patients to continue with, e.g., the exercises they have learned during physiotherapy, we consider the risk that it affected the subgroup analyses to be low, as we don't see why the prevalence of co-interventions would have been different between groups. Outcome completeness was imperfect, with paired pre- and post-treatment VAS data available for 61 of 81 feet. Later follow-up partly relied on telephone assessment, which introduces potential recall and reporting bias for toxicity. Follow-up was also relatively short and skewed, with a median of three months after the last course, which limits conclusions on durability. In addition, variation in providers, radiation technique, fractionation, and number of courses improves real-world relevance but complicates causal interpretation. Finally, the absence of observed toxicity is reassuring but cannot exclude rare but potentially catastrophic late effects, such as tumor induction. Current reviews suggest that radiation-induced cancer risk after modern benign-disease protocols is small, especially in older adults, yet it is not zero and remains part of the risk-benefit discussion [12,13].

Future work should now focus on identifying which patients benefit most. Multicenter prospective registries, pragmatic trials, or pooling of patient-level data from existing studies, like we provide for this one, could accomplish this. A particularly valuable next step would be the development of patient-level prediction models that integrate variables like age, baseline pain, symptom duration, prior therapies, imaging phenotype, and comorbidities. Longer-term safety follow-up would also be important for younger patients, even if the absolute carcinogenic risk with current protocols is thought to be low.

## Conclusions

In conclusion, the present study adds useful real-world evidence that low-dose radiotherapy can provide pain relief for a meaningful proportion of patients with refractory achillodynia, without observed toxicity in the present series. The magnitude and pattern of response are consistent with the best available achillodynia-specific studies and with current low-dose RT recommendations for painful degenerative-inflammatory disorders. Although the short median follow-up and uncontrolled design preclude definitive conclusions, the data strengthen the case for LDRT as a reasonable option after failure, intolerance, or contraindication of conservative treatment.
